# Comparable outcomes with a potential functional advantage of PCL preservation in medial‐stabilized total knee arthroplasty: A systematic review and meta‐analysis from the FP‐UCBM Knee Study Group

**DOI:** 10.1002/jeo2.70455

**Published:** 2025-11-14

**Authors:** Augusto Ferrini, Giuseppe Francesco Papalia, Giancarlo Giurazza, Andrea Tanzilli, Pietro Gregori, Edoardo Franceschetti, Rocco Papalia, Stefano Campi

**Affiliations:** ^1^ Operative Research Unit of Orthopeaedic and Trauma Surgery Fondazione Policlinico Universitario Campus Bio‐Medico Roma Italy; ^2^ Research Unit of Orthopaedic and Trauma Surgery, Department of Medicine and Surgery Università Campus Bio‐Medico di Roma Roma Italy; ^3^ Oncological Orthopaedics Department IFO – IRRCS Regina Elena National Cancer Institute Roma Italy

**Keywords:** mechanical alignment, medial Stabilized, posterior cruciate ligament‐retaining, posterior cruciate ligament‐substituting, total knee arthroplasty

## Abstract

**Purpose:**

The management of the posterior cruciate ligament (PCL) in medial‐stabilized (MS) total knee arthroplasty (TKA) remains a topic of debate. This systematic review and meta‐analysis investigate whether preserving or substituting the PCL in mechanical alignment (MA) MS‐TKA impacts clinical, functional and radiographic outcomes.

**Methods:**

Systematic literature searches (PubMed‐Medline, Scopus and Web of Science) followed PRISMA guidelines. Studies comparing PCL‐retaining (PCL‐r) and PCL‐substituting (PCL‐s) approaches in primary MS‐TKA with MA were included. Outcomes analyzed included range of motion (ROM), implant survivorship, patient‐reported outcome measures (PROMs), complication rates and radiological outcomes. Meta‐analyses were performed using Review Manager (RevMan) software 5.4.

**Results:**

Overall, seven studies involving 1376 knees met the inclusion criteria. No significant differences were observed between PCL‐r and PCL‐s cohorts regarding ROM, radiographic outcome or PROMs such as the Forgotten Joint Score, Oxford Knee Score and Knee Society Score (KSS‐Knee). A statistically significant difference was observed in KSS‐Function favouring the PCL‐r group (mean difference = −2.47, *p* = 0.008). Rates of complications and implant survivorship were comparable between the two techniques.

**Conclusion:**

Both PCL‐r and PCL‐s strategies yield largely comparable outcomes in MS‐TKA performed with MA. However, a significant difference in KSS‐Function favours the PCL‐r strategy, suggesting a potential advantage in functional recovery. The choice to retain or resect the PCL should be individualized based on patient characteristics, surgeon preference and implant design, but future studies are needed to explore the clinical implications of this functional benefit.

**Level of Evidence:**

Level III.

**Abbreviations**
HKA: hip–knee–ankle
KA: kinematic alignment
LDFA: lateral distal femoral angle
MA: mechanical alignment
MPTA: medial proximal tibial angle
PCL‐r: posterior cruciate ligament‐retaining
PCL‐s: posterior cruciate ligament‐substituting
PRISMA: Preferred Reporting Items for Systematic reviews and Meta‐Analyses
TKA: total knee arthroplasty

## INTRODUCTION

Total knee arthroplasty (TKA) is a widely performed surgical procedure aimed at restoring function and improving the quality of life in patients with end‐stage knee osteoarthritis (OA) [1, 6] [9, 10].

While cruciate‐retaining (CR) and posterior‐stabilized (PS) prostheses have long been the most common designs, medial stabilized (MS) implants have recently gained popularity, offering an additional alternative with clear biomechanical benefits [19].

MS‐TKA designs use a single‐radius curvature to better replicate natural knee kinematics [16] and enhanced medial compartment conformity [18]. However, the optimal management of the posterior cruciate ligament (PCL) in MS‐TKA remains unclear [2, 13, 32]. Some surgeons advocate for PCL sacrifice as it simplifies the surgical technique, reduces the risk of ligament imbalance and facilitates flexion‐extension gap balancing [3, 25]. Nonetheless, PCL sacrifice has different effects between individuals; studies show that using a thicker insert and reducing the tibial component's posterior slope were unsuccessful in restoring the loss of internal tibial rotation after PCL resection [27]. In contrast, others argue that preserving PCL improves proprioception and femoral rollback during flexion [12, 28]. Despite that, clinical evidence remains controversial [20, 21, 35], and PCL management often depends on surgeon preference [29]. We hypothesized that PCL preservation in MS‐TKA may result in superior functional outcomes compared to PCL substitution. The aim of this systematic review and meta‐analysis is to evaluate whether PCL preservation or resection in MS‐TKA leads to superior postoperative outcomes, including functional performance, complication rates and range of motion (ROM).

## MATERIALS AND METHODS

This systematic review and meta‐analysis included observational, prospective and retrospective studies. Eligible studies compared patients who underwent primary MS‐TKA with a mechanical alignment (MA) strategy, either with PCL resection (PCL‐s) or PCL preservation (PCL‐r). Included studies reported at least one of the following outcomes: implant survivorship, complications rate, ROM, patient‐reported outcome measures (PROMs), aseptic loosening, implant failure, revision surgery or functional outcomes. Studies were excluded if they included kinematic alignment (KA), non‐English language, systematic reviews, narrative reviews, meta‐analyses, case series, case reports or preclinical cadaveric studies.

### Search methods for identification of studies

The literature search was conducted in June 2025 across PubMed‐Medline, Scopus and Web of Science database using the following search strategy: ((‘posterior cruciate ligament’[MeSH Terms] OR ‘posterior cruciate ligament’[All Fields] OR PCL[All Fields]) AND (retain*[All Fields] OR preserv*[All Fields] OR sacrific*[All Fields] OR substitut*[All Fields]) AND (‘medial pivot’[All Fields] OR ‘medial congruent’[All Fields] OR ‘medial stabilized’[All Fields] OR ‘medial conformity’[All Fields]) AND (‘total knee arthroplasty’[MeSH Terms] OR ‘total knee replacement’[All Fields] OR TKA[All Fields])). The reference list of the articles was screened manually. Two independent reviewers screened titles and abstracts to identify eligible studies. A manual search of the bibliography of each published study was performed in order to find relevant articles that could potentially have been missed.

### Data collection, analysis and outcomes

Data extraction was independently performed by two reviewers. Following study characteristics were extracted: study characteristics such as authors, year of publication, study design, implant type, number of knees, patient demographics, such as sex, mean follow‐up, mean age, body mass index (BMI), diagnosis and radiological parameters, including hip–knee–ankle (HKA) angle [11], femoro‐tibial (FT) angle, anatomical medial proximal tibial angle (aMPTA), anatomical lateral distal femoral angle (aLDFA), femoral medial angle (FMA), tibial medial angle (TMA), slope, femoral flexion angle and radiolucent lines. Complications analyzed included instability, aseptic/septic loosening, anterior knee pain, periprosthetic fracture and infections. Clinical outcomes included ROM and PROMs such as Forgotten Joint Score (FJS), Knee Society Score (KSS), visual analogue scale (VAS), Western Ontario and McMaster Universities Osteoarthritis Index, Oxford Knee Score (OKS) and Knee Injury and Osteoarthritis Outcome Score (KOOS).

### Methodological quality assessment

Two reviewers used the Methodological Index for Non‐randomized Studies (MINORS) score to assess the quality of the included non‐randomized studies (Table 1). Meta‐analysis were conducted using Review Manager (RevMan) software 5.4. Postoperative clinical outcomes were compared between PCL‐r and PCL‐s and were expressed as continuous data with mean difference (MD) and 95% confidence intervals (95% CIs). Statistical heterogeneity was assessed using the *I* ^2^ test: a random‐effect model was adopted for data at high heterogeneity (*I* ^2^ > 60%). Statistical significance was set at *p* < 0.05.

**Table 1:** MINORS.

Study	Stated aim	Inclusion of patients	Collection of data	End points appropriate to the aim	Unbiased assessment of the study end point	Follow‐up	Loss to follow‐up less than 5%	Prospective calculation of the study size	An adequate control group	Contemporary groups	Baseline equivalence of groups	Adequate statistical analysis
Bae et al. [2]	2	2	2	2	2	2	2	2	2	2	2	2
Budhiparama et al. [5]	2	2	2	2	0	2	2	2	2	2	2	2
Hu et al. [15]	2	2	2	2	1	2	2	2	2	2	2	2
Foong et al. [8]	2	2	2	2	1	2	2	2	2	2	2	2
Macheras et al. [24]	2	2	2	2	1	2	2	2	2	2	2	2
Rossi et al. [31]	2	2	2	2	1	2	2	2	2	2	2	2
Ueyama et al. [33]	2	2	2	2	1	2	2	2	2	2	2	2

Abbreviation: MINORS, Methodological Index for Non‐randomized Studies.

## RESULTS

### Study research

**Figure 1:**
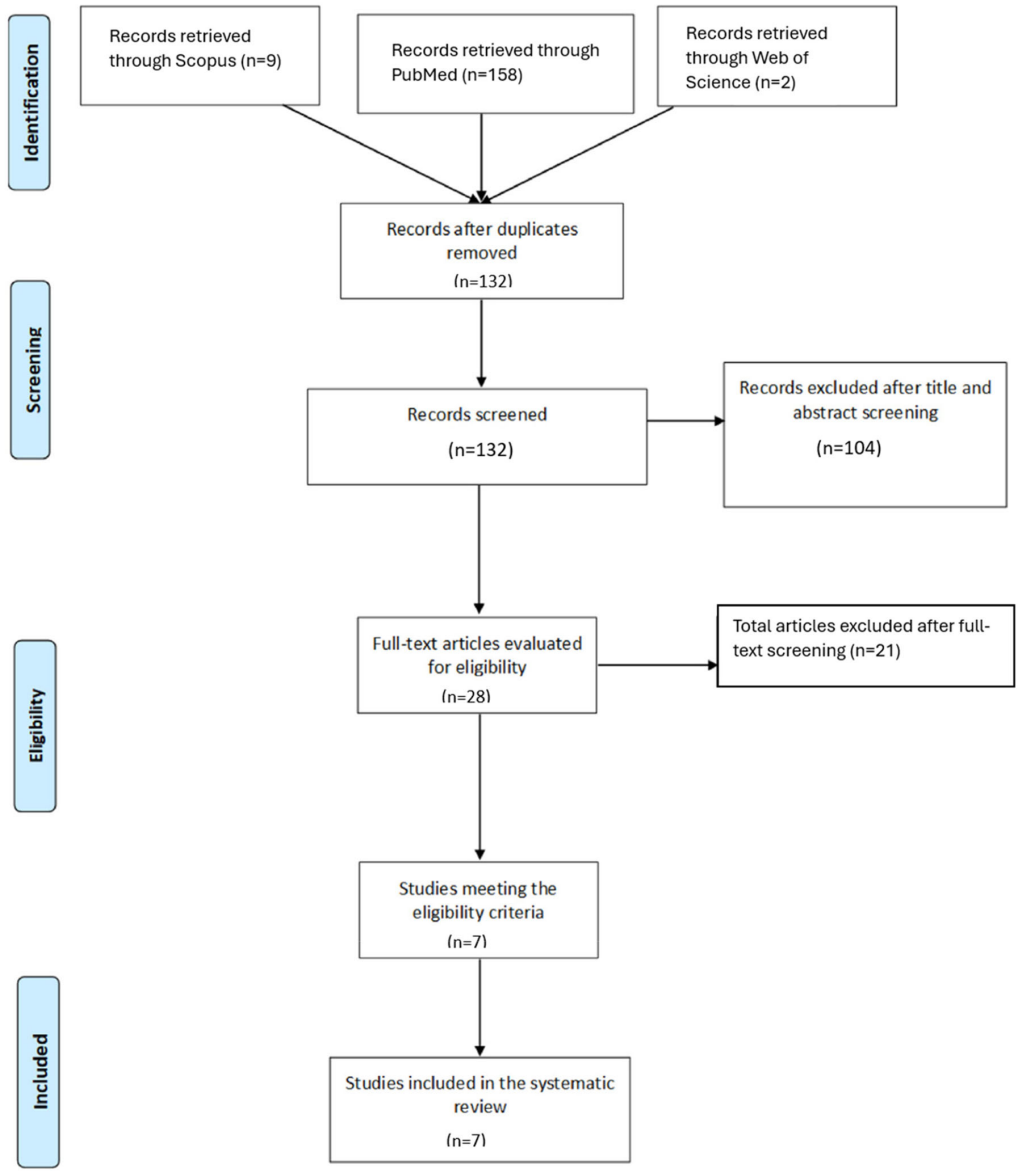
PRISMA search flow diagram. PRISMA, Preferred Reporting Items for Systematic Reviews and Meta‐Analyses.

The literature search identified 169 articles. After removing duplicates, 132 articles were screened based on their titles and abstracts. One hundred four studies were excluded due to irrelevance, inappropriate study design, lack of a comparison arm, non‐English language or being a meta‐analysis or systematic review. Twenty‐eight articles were selected for full‐text review. Finally, seven articles were deemed eligible for inclusion in this study. Among them, six were retrospective studies, and one was a prospective randomized controlled trial (Figure 1).

### Demographic data

A total of 1376 knees were included, with 548 in the PCL‐r group and 828 in the PCL‐s group, all performed with standard MA. The mean patient age was 70.5 years (range: 65.8–80). The PCL‐s group had a mean age of 70.7 years, with 80% female, while the PCL‐r group had a mean age of 70 years (range: 67.5–74.6), with 73.4% female. Mean BMI data were reported in seven studies: in the PCL‐s group, the mean value was 27.2 (range: 23.4–30.1); while in the PCL‐r group, it was 27.1 (range: 23.3–29). The surgical indications were OA (95.1% in the PCL‐s group vs. 94.8% in the PCL‐r group), followed by rheumatoid arthritis (3.9% in the PCL‐s group vs. 3.7% in the PCL‐r group) and avascular necrosis. The mean follow‐up across studies was 6 years, ranging from 1 year to 15.2 years, calculated as the arithmetic mean of each study's follow‐up durations, weighted by sample size. Rossi et al. [31] used the Persona Cruciate‐Retaining Medial Congruent Knee in all patients. Foong et al. [8] included only patients receiving the Persona MC system. Implant details were not specified by other authors.

Table 2 summarizes study characteristics and patient demographics.

**Table 2:** Study characteristics and patient demographics.

Study	Year	Type of study	Level of evidence	Group	Implant	No. of knee	Sex	BMI	Deformity	Mean FUP	Mean age of surgery	Diagnosis
Bae et al. [2]	2011	RSC	III	PCL‐s	MP	70	54F 6M	26.7 ± 3.3	NR	3.9	65.8 ± 9.0	65 Primary osteoarthritis, 2 rheumatoid arthritis, 3 avascular necrosis
				PCL‐r	MP	67	58F 1M	26.09 ± 2.9	NR		67.5 ± 4.6	61 Primary osteoarthritis, 3 rheumatoid arthritis, 3 avascular necrosis
Budhiparama et al. [5]	2023	RCT	I	PCL‐s	MP	33	24F 9M	25.8	33 Varus	2 (33m ± 7.1)	71	33 Osteoarthritis
				PCL‐r	MP	33		25.8	33 Varus		71	33 Osteoarthritis
Foong et al. [8]	2024	RSC	III	PCL‐s	Persona MC	50	37F 13M	28	50 Varus knee	1	66	50 Osteoarthritis
				PCL‐r		26	20F 6M	29	26 Varus knee	1	67	26 Osteoarthritis
Hu et al. [15]	2023	RSC	III	PCL‐s	CS‐MP	168	NR	28.1 ± 3.9	NR	8.7 ± 0.9	67.5 ± 7.5	168 Osteoarthritis
				PCL‐r	CR‐MP	84	NR	27.9 ± 3.3	NR	8.6 ± 1.3	68.2 ± 7.3	84 Osteoarthritis
Macheras et al. [24]	2017	RSC	III	PCL‐s	MP	165	99F 50M	30.1	NR	15.2	80	116 Primary osteoarthritis, 18 rheumatoid arthritis, 14 post‐traumatic arthritis, 1 avascular necrosis
				PCL‐r	MP	182	116F 60M	28.9	NR		73	142 Primary osteoarthritis, 15 rheumatoid arthritis, 17 post‐traumatic arthritis, 2 avascular necrosis
Rossi et al. [31]	2023	RSC	III	PCL‐s	Persona CR‐MC	85	100F 61M	28.5 ± 5.2	NR	6 (72m ± 12)	68.6 ± 9	NR
				PCL‐r		80			NR			NR
Ueyama et al. [33]	2022	RSC	III	PCL‐s	CS‐MP	257	241F 16M	23.4 ± 3.4	2 Valgus knee, 28 neutral knee, 227 varus knee	10.1 ± 1.7	76.2 ± 7.3	244 Osteoarthritis, 10 rheumatoid arthritis, 3 avascular necrosis
				PCL‐r	CR‐MP	76	67F 9M	23.3 ± 2.9	2 Valgus knee, 9 neutral knee, 65 varus knee	10 ± 1.7	74.6 ± 7.3	74 Osteoarthritis, 0 rheumatoid arthritis, 2 avascular necrosis

Abbreviations: BMI, body mass index; PCL‐r, posterior cruciate ligament‐retaining; PCL‐s, posterior cruciate ligament‐substituting; RCT, randomised controlled trial; RSC, retrospective cohort study.

### Radiological and clinical outcomes

**Figure 2:**
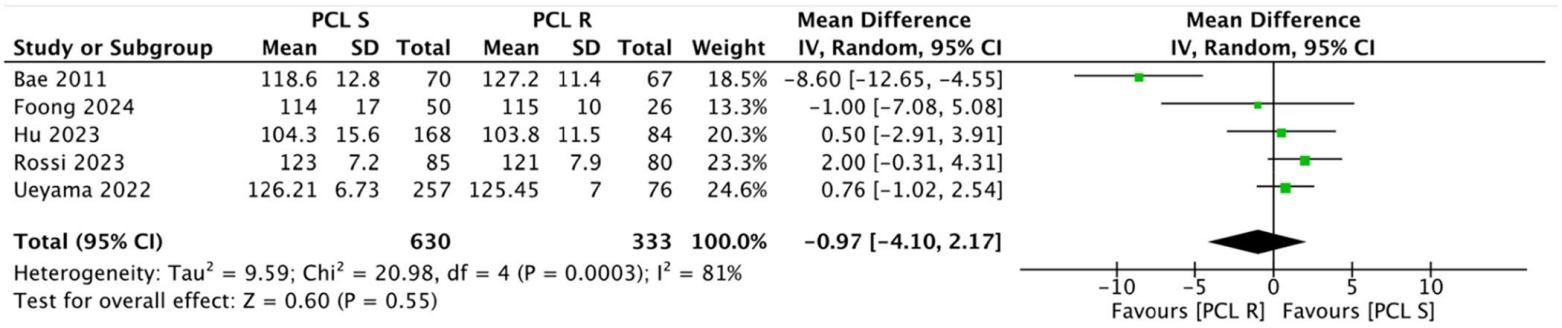
Forest plot of the meta‐analysis of five studies assessing the range of movement with mean and standard deviation (SD) in the two groups. CI, confidence interval; IV, intravenous; PCL‐r, posterior cruciate ligament‐retaining; PCL‐s, posterior cruciate ligament‐substituting.

**Figure 3:**
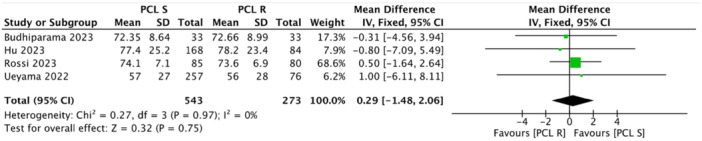
Forest plot of the meta‐analysis of four studies assessing the Forgotten Joint Score with mean and standard deviation (SD) in the two groups. CI, confidence interval; IV, intravenous; PCL‐r, posterior cruciate ligament‐retaining; PCL‐s, posterior cruciate ligament‐substituting.

**Figure 4:**
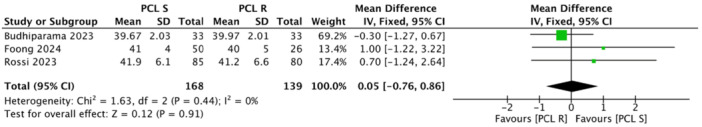
Forest plot of the meta‐analysis of three studies assessing the Oxford Knee Score with mean and standard deviation (SD) in the two groups. CI, confidence interval; IV, intravenous; PCL‐r, posterior cruciate ligament‐retaining; PCL‐s, posterior cruciate ligament‐substituting.

**Figure 5:**
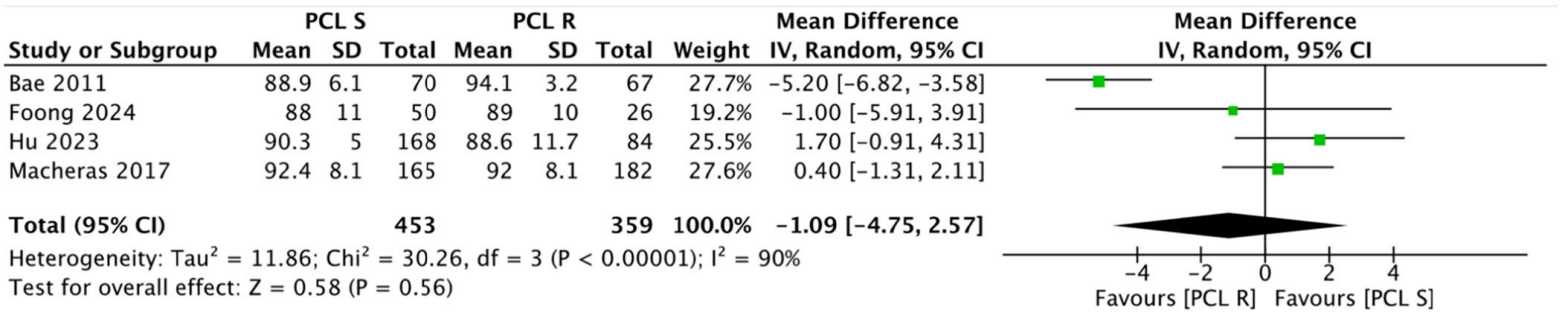
Forest plot of the meta‐analysis of four studies assessing the Knee Society Score with mean and standard deviation (SD) in the two groups. CI, confidence interval; IV, intravenous; PCL‐r, posterior cruciate ligament‐retaining; PCL‐s, posterior cruciate ligament‐substituting.

**Figure 6:**
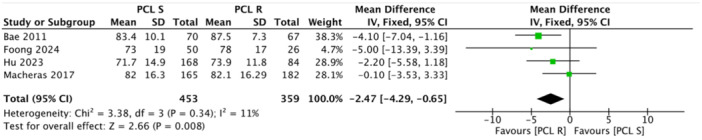
Forest plot of the meta‐analysis of four studies assessing the Knee Society Score with mean and standard deviation (SD) in the two groups. CI, confidence interval; IV, intravenous; PCL‐r, posterior cruciate ligament‐retaining; PCL‐s, posterior cruciate ligament‐substituting.

Multiple studies reported no significant differences in radiological parameters between the PCL‐r and PCL‐s groups, particularly regarding postoperative limb alignment and femoral‐tibial angles [2, 5, 15, 33]. For instance, Bae et al. [2] and Budhiparama et al. [5] found no difference in postoperative limb alignment between the PCL‐r and PCL‐s groups. Foong et al. [8] reported only post‐operative femoral tibial angle (3.46 ± 3.58 in the PCL‐s group and 5 ± 3.05 in the PCL‐r group). No significant differences were found in the radiographic outcomes in the retrospective study by Hu et al. [15]. Between the PCL‐s group and PCL‐r group, the preoperative FTA (−3.8 ± 6.0 vs. −3.9 ± 4.9, *p* = 0.997), postoperative FTA (3.7 ± 3.0 vs. 3.5 ± 3.7, p = 0.646), aLDFA (84.4 ± 2.3 vs. 85.0 ± 3.3, *p* = 0.094) and aMPTA (88.2 ± 2.4 vs. 88.2 ± 2.4, *p* = 0.970) were all within the normal range and comparable. Macheras et al. [24] evaluated the mean femoral medial angle (95°) (92–101°), the mean femoral flexion (1°) (−2° to 4°) and the mean tibial medial angle (88.5°) (range: 87–92°) at final follow‐up. Rossi et al. [31] did not report statistical difference in radiological measurement between the two groups at final follow‐up, but for the tibial slope. A retrospective study by Ueyama et al. [33] reported a change in HKA angle from 183.8° to 176.2° in the PCL‐r group and from 182.7° to 176.1° in the PCL‐s group. Posterior tibial slope (PTS) was also similar in most studies. Hu et al.: [15] PTS was 4.7  ±  1.7° in the PCL‐s group and 4.4  ±  1.4° in the PCL‐r group (*p* = 0.243). However, one study found a significant difference in tibial slope between the two groups [31] (Table 3). No study reported progressive or symptomatic radiolucent lines more than 2 mm suggestive of aseptic loosening (Table 4). Overall results showed comparable outcomes except for KSS‐Function. The pooled analysis across five studies did not show any difference in ROM (MD = −0.97, 95% CI = −4.10 to 2.17, *p* = 0.55) (Figure 2). This finding had minimal variability and overlapping CI among the included studies. In terms of ROM, no study reported a clinically significant advantage for either technique. Meta‐analysis also demonstrated no statistical differences in FJS (MD = 0.29, 95% CI = − 1.48 to 2.06, *p* = 0.75) (Figure 3), OKS (MD = 0.05, 95% CI = −0.76 to 0.86, *p* = 0.91) (Figure 4) and KSS Knee (MD = −1.09, 95% CI = −4.75 to 2.57, *p* = 0.56) (Figure 5). However, the pooled analysis of four studies demonstrated a statistically significant improvements in KSS Function with higher results in the PCL‐r group (MD = −2.47, 95% CI = −4.29 to −0.65, *p* = 0.008) (Figure 6) (Table 5). Although the MD did not exceed the minimal clinically important difference (MCID), this finding suggests a potential functional advantage associated with PCL retention in MS‐TKA.

**Table 3:** Radiological outcomes.

Study	HKA pre	HKA post	FT angle pre	FT angle post	aMPTA pre	aMPTA post	aLDFA pre	aLDFA post	FMA post	TMA post	Femoral flexion (*γ* angle)	SLOPE pre	SLOPE post	Radiolucent line
Bae et al. [2]	NR	NR	Varus 4.0 ± 4.5	Valgus 5.5 ± 2.8	NR	NR	NR	NR	96.0 ± 2.7	89.1 ± 2.0	2.5 ± 1.7	NR	5.7° (84.3 ± 3.2)	NR
	NR	NR	Varus 4.2 ± 4.3	Valgus 6.0 ± 2.7	NR	NR	NR	NR	95.2 ± 2.2	90.8 ± 1.9	3.6 ± 2.3	NR	6° (84.0 ± 2.4)	NR
Budhiparama et al. [5]	188.51 ± 3.53	181.30 ± 2.13	NR	NR	NR	NR	NR	NR	NR	NR	NR	NR	NR	NR
	188.36 ± 2.93	180.27 ± 2.25	NR	NR	NR	NR	NR	NR	NR	NR	NR	NR	NR	NR
Foong et al. [8]	NR	NR	NR	3.46 ± 3.58	NR	NR	NR	NR	NR	NR	NR	NR	NR	NR
	NR	NR	NR	5 ± 3.05	NR	NR	NR	NR	NR	NR	NR	NR	NR	NR
Hu et al. [15]	NR	NR	−3.8 ± 6.0	3.7 ± 3.0	NR	88.2 ± 2.4	NR	84.4 ± 2.3	NR	NR	NR	NR	4.7 ± 1.7	NR
	NR	NR	−3.9 ± 4.9	3.5 ± 3.7	NR	88.2 ± 2.4	NR	85.0 ± 3.3	NR	NR	NR	NR	4.4 ± 1.4	NR
Macheras et al. [24]	NR	NR	NR	NR	NR	NR	NR	NR	95°	88.5°	1°	NR	NR	NR
	NR	NR	NR	NR	NR	NR	NR	NR				NR	NR	NR
Rossi et al. [31]	NR	177.2 ± 3.1°	NR	NR	NR	NR	NR	NR	93.5 ± 2.3°	88.9 ± 1.3°	2.7 ± 2.1°	NR	4.8° (83.3 ± 2.4°)	3 (3.5%)
	NR	177.6 ± 3.1°	NR	NR	NR	NR	NR	NR	92.7 ± 2.6°	89.6 ± 1.2°	2.9 ± 2.3°	NR	6.7° (85.2 ± 1.4°)	4 (5%)
Ueyama et al. [33]	183.8 (169.9–195.4)	176.2 (172.9–179.8)	NR	NR	NR	NR	NR	NR	94.1°	90.7°	2.7°	NR	4°	29 (11.3%)
	182.7 (170.0–192.1)	176.1 (173.6–178.7)	NR	NR	NR	NR	NR	NR	94°	90.4°	2.4°	NR	4.2°	8 (10.8%)

Abbreviations: aLDFA, anatomical lateral distal femoral angle; aMPTA, anatomical medial proximal tibial angle; FMA, femoral medial angle; FT, femoro‐tibial; HKA, hip–knee–ankle; NR, not reported; TMA, tibial medial angle.

**Table 4:** Complications.

Study	Instability	Aseptic loosening	Knee clicking	Anterior knee pain	Septic loosening	Periprosthetic fracture	Infection (surgical site, pji)	Reoperation
Bae et al. [2]	NR	1	NR	NR	NR	2	NR	1 ORIF, 1 revision TKA
	NR	NR	NR	NR	NR	1	2	1 (two‐stage revision), 1 ORIF
Budhiparama et al. [5]	0	0	NR	NR	0	0	0	0
	0	0	NR	NR	0	0	0	0
Foong et al. [8]	NR	NR	NR	NR	NR	NR	NR	NR
	NR	NR	NR	NR	NR	NR	NR	NR
Hu et al. [15]	2 (1.2%)	NR	4 (2.4%)	3 (1.8%)	NR	NR	4 (2.4%)	NR
	0	NR	2 (2.4%)	2 (2.4%)	NR	NR	NR	NR
Macheras et al. [24]	NR	NR	NR	3	NR	1	3	NR
	NR	NR	NR		NR			NR
Rossi et al. [31]	NR	NR	NR	NR	NR	NR	1 (1.18%)	NR
	NR	NR	NR	NR	NR	NR	1 (1.25%)	NR
Ueyama et al. [33]	4 (1.55%)	0	NR	NR	2 (0.77%)	3 (1.7%)	1 (0.4%)	3 Exchange insert, 2 Revision of tibial components to constraint, 2 ORIF, 2 Two‐stage revision of total components, 1 DAIR
	2 (2.63)	0	NR	NR	0	2 (2.63%)	0	2 Exchange insert, 1 revision of tibial components to constrain, 1 ORIF

Abbreviations: DAIR, debridement, antibiotics and implant retention; NR, not reported; ORIF, open reduction and internal fixation; TKA, total knee arthroplasty.

**Table 5:** Clinical outcomes.

Study	FJS pre	FJS	ROM pre	ROM post	KSS knee score pre	KSS knee score post	KSS function score pre	KSS function score post	KNEE extension pre	KNEE extension post	KNEE flexion pre	KNEE flexion post	WOMAC pre	WOMAC post	OKS pre	OKS post	KOOS‐S pre	KOOS‐S post	KOOS‐qol pre	KOOS‐qol post	VAS pre	VAS post	Survival rate
Bae et al. [2]	NR	NR	110.1 ± 18.6°	118.6 ± 12.8°	58.4 ± 9.6	88.9 ± 6.1	52.4 ± 7.7	83.4 ± 10.1	NR	NR	NR	NR	NR	NR	NR	NR	NR	NR	NR	NR	NR	NR	NR
	NR	NR	119.3 ± 12.3°	127.2 ± 11.4°	60.9 ± 4.7	94.1 ± 3.2	54.8 ± 6.5	87.5 ± 7.3	NR	NR	NR	NR	NR	NR	NR	NR	NR	NR	NR	NR	NR	NR	NR
Budhiparama et al. [5]	NR	72.35 ± 8.64	NR	NR	NR	NR	NR	NR	NR	NR	NR	NR	NR	NR	23.30 ± 3.3	39.67 ± 2.03	41.03 ± 10.49	84.19 ± 3.57	25.48 ± 9.79	82.75 ± 4.70	8.09 ± 0.98	2.00 ± 0.71	NR
	NR	72.66 ± 8.99	NR	NR	NR	NR	NR	NR	NR	NR	NR	NR	NR	NR	23.88 ± 3.29	39.97 ± 2.01	40.76 ± 10.57	84.41 ± 3.77	25.24 ± 7.96	82.94 ± 4.76	7.91 ± 0.80	1.94 ± 0.79	NR
Foong et al. [8]	NR	NR	107 ± 19	114 ± 17	53 ± 17	88 ± 11	49 ± 22	73 ± 19	NR	NR	NR	NR	NR	NR	27 ± 8	41 ± 4	NR	NR	NR	NR	NR	NR	NR
	NR	NR	102 ± 19	115 ± 10	54 ± 18	89 ± 10	57 ± 24	78 ± 17	NR	NR	NR	NR	NR	NR	26 ± 7	40 ± 5	NR	NR	NR	NR	NR	NR	NR
Hu et al. [15]	NR	77.4 ± 25.2	84.9 ± 16.1	104.3 ± 15.6	23.4 ± 6.1	90.3 ± 5.0	34.2 ± 15.3	71.7 ± 14.9	NR	NR	NR	NR	74.6 ± 10.9	12.6 ± 13.9	NR	NR	NR	NR	NR	NR	NR	NR	98.6%
	NR	78.2 ± 23.4	84.9 ± 12.3	103.8 ± 11.5	23.7 ± 4.6	88.6 ± 11.7	34.4 ± 14.6	73.9 ± 11.8	NR	NR	NR	NR	74.9 ± 7.3	11.4 ± 13.4	NR	NR	NR	NR	NR	NR	NR	NR	100%
Macheras et al. [24]	NR	NR	86°	121°	32.5 ± 16.3	92.4 ± 8.1	42.7 ± 13.0	82.0 ± 16.3	NR	NR	NR	NR	31.0 ± 10.3	79.4 ± 17.2	44.7 ± 5.0	22.0 ± 8.8	NR	NR	NR	NR	NR	NR	98.2%
	NR	NR	84°	118°	32.6 ± 17.6	92.0 ± 8.1	42.8 ± 12.9	82.1 ± 16.29	NR	NR	NR	NR	30.5 ± 9.8	79.2 ± 17.5	44.4 ± 5.2	21.9 ± 9.5	NR	NR	NR	NR	NR	NR	
Rossi et al. [31]	NR	74.1 ± 7.1	NR	123 ± 7.2	100 ± 5	163 ± 4.4	NR	NR	NR	NR	NR	NR	NR	NR	20.9 ± 0.9	41.9 ± 6.1	NR	NR	NR	NR	NR	NR	100%
	NR	73.6 ± 6.9	NR	121 ± 7.9	102 ± 3	166 ± 4.2	NR	NR	NR	NR	NR	NR	NR	NR	22.2 ± 0.8	41.2 ± 6.6	NR	NR	NR	NR	NR	NR	100%
Ueyama et al. [33]	NR	57 (SD 27; 35–75)	111.21 ± 10.53	126.21 ± 6.73	39.7 (28–55)	87.8 (75–100)	41.5 (35–60)	90.3 (75–100)	−2.4° (−20 to 0)	−0.1° (−10 to 5)	114° (90–135)	118° (85–135)	NR	NR	NR	NR	NR	NR	NR	NR	8.0 (4–10)	1.6 (0–4)	95.8% with reoperation
	NR	56 (SD 28; 30–76)	111.97 ± 11.59	125.45 ± 7.00	39.1 (30–51)	87.0 (74–95)	41.8 (35–50)	90.0 (745–95)	−2.9° (−20 to 0)	−0.2° (−10 to 5)	113° (85–135)	116° (85–135)	NR	NR	NR	NR	NR	NR	NR	NR	8.2 (4–10)	1.6 (0–4)	

Abbreviations: FJS, Forgotten Joint Score; KSS, Knee Society Score; NR, not reported; OKS, Oxford Knee Score; SD, standard deviation; VAS, visual analogue scale; WOMAC, Western Ontario and McMaster Universities Osteoarthritis Index.

## DISCUSSION

The main findings of this systematic review and meta‐analysis indicate that clinical outcomes, including PROMs, as well as knee kinematics, are largely comparable between the PCL‐r and PCL‐s MS‐TKA groups [17, 25], with the notable exception of a statistically significant improvement in KSS Function score favouring PCL retention. Both implant designs demonstrated high patient satisfaction and similar survivorship rates [22]. A previous study by D'Ambrosi et al. [7] comparing CR and PS TKA showed no differences in sport‐related PROMs, ROM, pain, return‐to‐sport rates or implant longevity at 5‐year follow‐up. Likewise, Ueyama et al. [33] and Rossi et al. [31] reported no differences in ROM or functional outcomes between PCL‐r and PCL‐s in over follow‐ups extending up to 10 years. A prospective series also found no significant differences between PCL‐r and PCL‐s in ROM, PROMs or implant survivorship over 6–9 years [15]. Similarly, studies comparing simultaneous bilateral MS‐TKA, where one knee retained the PCL and the contralateral knee sacrificed it, reported no differences in ROM, PROMs or radiographic outcomes [5]. Radiographic equivalence may suggest similar alignment outcomes, but dynamic biomechanical equivalence of both approaches during gait or flexion‐extension movements cannot be determined [5]. While our findings are mostly aligned with the literature, our meta‐analysis revealed a statistically significant improvement in the KSS Function in favour of PCL retention. Some authors suggest potential proprioceptive benefits from PCL retention, although clinical evidence remains limited and inconclusive [4, 30]. Despite not exceeding the MCID threshold, this result raises important considerations regarding proprioception and neuromuscular function potentially conferred by preserving the PCL. This result aligns with recent evidence suggesting better gait and functional scores in PCL‐r patients and should prompt further exploration in subgroups with high functional demands or preserved ligament integrity [26]. The relevance of PCL retention may be amplified when an alignment strategy maintains its physiological tension and function [14]. The lack of differences in outcomes with MA is likely due to the ‘one‐size‐fits‐all’ approach, which does not account for native knee biomechanics and kinematics, potentially obscuring the benefits of PCL retention. However, we excluded the retrospective study by Giustra et al. [13] due to its focus on KA, which differed from our approach. Their comparison of PCL‐retaining and PCL‐substituting implants in total knee arthroplasty using an MS design did not find significant differences in postoperative ROM, PROMs or radiographic findings in varus knees. In a cadaveric study, Nedopil et al. [26] demonstrated increased passive internal tibial rotation with PCL retention compared to PCL excision, with mean values of 15° and 7°, respectively, between full extension and 90° flexion, with a negligible risk of anterior lift‐off. Nevertheless, in vivo studies with larger sample sizes are necessary to determine whether PCL retention provides clear advantages in MS‐TKA performed with unrestricted calliper‐verified KA. Future research focusing on specific patient subgroups may elucidate differential responses and potential benefits in selected populations. The preservation of the PCL in MS implants has been associated with theoretical benefits, such as improved tibial rotation and more natural tibial slope reproduction, as demonstrated in cadaveric studies and kinematic assessments [26]. However, clinical studies have shown that these biomechanical advantages do not consistently correlate with superior clinical outcomes. Modern PCL‐s designs have addressed the kinematic alterations through tibial slope adjustments and congruent inserts, ensuring stability without compromising functional outcomes [23]. Nonetheless, some studies have noted marginal improvements in ROM during deep flexion with PCL‐s designs, attributed to cam‐post mechanisms or high‐congruency inserts. Anyway, these differences did not translate into substantial functional benefits. Finally, the role of the PCL in proprioception remains a subject of debate [34].

### Limitations

A few limitations should be considered in interpreting these findings. Notably, considerable heterogeneity was present across the included studies in terms of design, outcome measures and evaluation methods. In addition, most patients included in the studies were female, which may limit the generalizability of the findings to male populations and should be considered when interpreting the results. Moreover, the relatively short mean follow‐up period does not allow definitive conclusions regarding potential long‐term differences in radiological outcomes, implant loosening or survivorship.

## CONCLUSION

PCL‐r and PCL‐s MS‐TKA performed with MA provide comparable overall outcomes. However, a small but statistically significant improvement in the KSS Function score was observed in the PCL‐r group, suggesting a potential functional advantage of PCL preservation. Further long‐term studies, focusing on different alignment strategies and specific patient subgroups, are needed to better clarify the role of PCL preservation in TKA.

## AUTHOR CONTRIBUTIONS

Augusto Ferrini was responsible for conceptualization and writing of the manuscript. Stefano Campi and Edoardo Franceschetti supervised data acquisition and were responsible for formal analysis. Giancarlo Giurazza, Andrea Tanzilli and Giuseppe Francesco Papalia were responsible for data acquisition, extraction and visualization. Pietro Gregori qualified as the corresponding author. Rocco Papalia was responsible for reviewing and editing the manuscript. All authors have given final approval of the version to be published.

## CONFLICT OF INTEREST STATEMENT

The authors declare no conflicts of interest.

## ETHICS STATEMENT

The ethics statement is not available.

## Data Availability

The data that support the findings of this study are available from the corresponding author, P.G., upon reasonable request.
